# Spinning of Polysulfone Hollow Fiber Membranes Using Constant Dope Solution Composition: Viscosity Control via Temperature

**DOI:** 10.3390/membranes12121257

**Published:** 2022-12-12

**Authors:** Dmitry Matveev, Ilya Borisov, Vladimir Vasilevsky, Galina Karpacheva, Vladimir Volkov

**Affiliations:** A.V. Topchiev Institute of Petrochemical Synthesis RAS, Russian Academy of Sciences, 119991 Moscow, Russia

**Keywords:** hollow fiber membrane, dope solution, viscosity effect, temperature control, spinning parameters, phase inversion kinetics, polysulfone, gas permeability

## Abstract

The dope solution viscosity is an important parameter that largely determines the properties of hollow fiber membranes. In the literature available today, the change in viscosity is carried out only by changing the quantitative and/or qualitative dope solution compositions. However, such an important spinning parameter as temperature should significantly affect the dope solution viscosity. For the first time, the influence of the dope solution viscosity of a constant composition on polysulfone hollow fiber membrane properties was studied. The hollow fiber membranes were obtained by the phase separation method induced by a non-solvent (NIPS). The change in the dope solution temperature was carried out in the temperature range of 17–27 °C, providing a dope solution viscosity range of 34.3–21.6 Pa∙s. This work shows that even in such a narrow temperature range, the properties of polysulfone hollow fiber membranes change significantly. With a decrease in the viscosity in this temperature range, the wall thickness of the hollow fiber membrane decreases by 2.8 times; the permeance for the individual gases He and CO_2_ increases by 1.6–1.8 times, respectively; the ideal selectivity decreases by 1.12 times; the mean flow pore size increases by 1.63 times; and the surface porosity increases about 3 times.

## 1. Introduction

Hollow fiber membranes have a number of advantages compared to flat-sheet membranes, primarily due to the high packing density of the membrane per unit volume of the module [1,2,3]. A lot of membrane processes use asymmetric hollow fiber membranes. A porous asymmetric membrane is an anisotropic structure with a thin, dense (finely porous) skin layer located on a porous substrate of the same material. Separation is achieved mainly by the skin layer of the asymmetric membrane, while the larger pore support provides mechanical strength. The skin layer has a thickness from several tens to hundreds of nanometers and performs separation with high productivity [3]. At present, the phase inversion method is the most common method for obtaining asymmetric hollow fiber membranes [2,4]. Phase inversion is a demixing process where the polymer is transformed from a liquid to a solid state. Phase separation can be caused by solvent evaporation (SE), thermal exposure (TIPS) and a non-solvent (NIPS). In turn, the production of hollow fiber membranes by NIPS can be carried out in three ways: dry, wet and a combination of dry and wet spinning methods.

The creation of hollow fiber membranes involves a large number of spinning parameters that can affect the thermodynamics and kinetics of phase inversion [4]. These factors, in turn, determine the morphology, geometry and transport properties of the resulting hollow fiber membrane. Such spinning parameters include the composition, viscosity and flow rate of the dope solution; the chemical composition, temperature and flow rate of the bore fluid; the air gap distance; the composition and temperature of external coagulation; the design and dimensional parameters of the spinneret; the draw ratio [2,4,5,6,7], etc.

The viscosity is a diffusional property of the dope solution, which affects the phase demixing rate [4,8]. This critical parameter can affect the phase inversion kinetics due to its influence on the mutual diffusion between the solvent and non-solvent [4,9]. In addition, high viscosity will promote the chain entanglement of a nascent hollow fiber [4,10]. Obviously, a change in the viscosity of the dope solution (ceteris paribus) can affect some other spinning parameters, such as the flow rate of the dope solution (cm^3^/s), the draw ratio, the spinning speed of the hollow fiber (cm/s), and therefore, the residence time in the air gap (s), etc. The viscosity of the polymeric solution plays a major role in the resulting morphology of the membranes obtained. We have previously shown that regardless of the acrylonitrile copolymer composition and solvent nature (DMF and DMSO), the studied membranes possessed a finger-like porous structure if the viscosity of the polymer solution was lower than 42 Pa∙s and a sponge-like porous structure at a viscosity greater than 78 Pa∙s [11].

Usually, the dope solution viscosity can be changed by the following ways: varying of the polymer concentration [11,12,13,14,15,16,17,18,19,20,21] and molecular weight [11,12,18]; the quantitative ratio of polymers [19,21,22] (if a polymer mixture is used); the composition and monomer unit ratio of copolymers [11]; the types of solvents [11]; the types [12,23], concentrations [12,20,24,25,26] and molecular weight [27] of the additives in the dope solution, etc. Table 1 presents a literature data analysis from the standpoint of the main ways to change the dope solution viscosity and the effect of the viscosity change on the properties of the resulting hollow fiber membranes. As follows from the table, in the overwhelming majority of cases, an increase in the dope solution viscosity leads to the following three patterns/trends: (1) the performance of hollow fiber membranes decreases, while the separating properties improve; (2) the pore size and porosity of the resulting membranes decrease; and (3) a change in membrane morphology from a finger-like to a sponge-like structure.

Unfortunately, in all works presented in Table 1 [13,14,15,16,17,18,19,20,21,22,23,24,25,26,27], along with the change in viscosity, the quantitative and/or qualitative composition of the dope solutions also changed. This does not make it possible to speak unambiguously about how the viscosity affects the properties of the resulting membranes. At the same time, such an important parameter of the spinning process as the temperature should significantly affect the dope solution viscosity while its composition stays unchanged. Ghasem et al. [28] studied the effect of different extrusion temperatures (140–170 °C) on the PVDF hollow fiber membrane properties produced by the TIPS method. The hollow fiber membrane was spun at a temperatures close to the PVDF melting point (177 °C). It has been shown that decreasing the dope solution viscosity leads to an increase in pore size, porosity and CO_2_ flux. In contrast to TIPS, the production of asymmetric hollow fiber membranes by the most common NIPS method is usually carried out at “room temperature” (20–25 °C) [13,14,17,18,20,21,22,23,24,25,26]. Moreover, quite often authors do not indicate the specific temperature regime of the spinning process and limit themselves to only indicating “at room temperature” [16,29,30]. 

To the surprise of the authors, there are no systematic studies on the influence of the dope solution temperature on the properties of hollow fiber membranes obtained by the NIPS method in the literature available today. Plisko et al. [31] and Ursino et al. [32] obtained hollow fibers from PSF and PESF, respectively, by the NIPS method at different dope solution temperatures. However, in these works, a series of samples of hollow fiber membranes were obtained by simultaneously changing both the temperature and the concentration of polymers in the spinning solution.

PSF is a widespread membrane material and is widely used for hollow fiber membrane production [1,2]. Due to its low cost, high thermal and mechanical stability and high chemical resistance, this polymer occupies a leading position in membrane science and technology [33].

With this in mind, the aim of this work was to investigate the effect of the dope solution viscosity on the morphology and transport properties of PSF hollow fiber membranes. It is important to emphasize that the dope solution viscosity was varied only by the temperature for the solutions of the same composition. Moreover, the temperature variation in the dope solution was carried out in a rather narrow temperature range of 17–27 °C, which is quite consistent with the usual spinning modes at so-called “room temperature”.

## 2. Materials and Methods

### 2.1. Materials

Polysulfone (BASF Ultrason^®^ S 6010, M_w_ = 68 kg/mol) was used in the form of granules in this work, as well as N-methylpyrrolidone (Acros Organics, Geel, Belgium, 99% extra pure) as a solvent for the dope solution preparation. Polyethylene glycol with an average molecular weight of 400 g∙mol^–1^ (PEG-400, Acros Organics) was utilized as a pore-forming additive. All reagents were used without additional purification.

### 2.2. The Dope Solution Preparation

To create the PSF hollow fiber membranes, the dope solution was chosen, which was used by us earlier [34,35,36,37]. PSF and PEG-400 (mass ratio 1:1.36) were placed in a thermostatically controlled reactor and stirred at a speed of 150 rpm at a temperature of 50 °C. Then, NMP was added to this system while increasing the stirring speed to 500 rpm. Under these conditions, the solution was mixed until homogeneity was achieved. The concentration of PSF in the dope solution assumed a value of 22 wt %. 

Before the hollow fiber membrane spinning, the polymer solution was filtered. The solution was heated to 40 °C in order to reduce its viscosity and, consequently, the filtration time, after which the dope solution was filtered under a nitrogen pressure of 1.8–2.0 bar through a stainless steel mesh (cutoff rating 4–5 μm). After the filtration procedure, the polymer solution was cooled to room temperature and degassed under vacuum overnight.

### 2.3. Measurement of the Dope Solution Viscosity

After preparing the dope solution, it was thermostated to the required temperature to determine the dynamic viscosity using a Brookfield viscometer Brookfield DV2T-RV. The measured temperature of the dope solutions varied in the range of 16–70 °C.

### 2.4. Hollow Fiber Membranes Preparation

The samples of PSF hollow fiber membranes were obtained using the setup described elsewhere [38]. The setup makes it possible to equalize the take-up speed of the receiving drum (handpicked) to the dope extrusion flow rate, thereby eliminating the draw ratio caused by winding [38]. In this way, the spinning speed can be determined. Before the spinning process, the room in which the spinning setup was located was thermostatically controlled using an air conditioning system. This made it possible to control the temperature of the whole spinning device as well as the dope solution and all other components of the spinning process with an accuracy of 0.5 °C in the temperature range of 17–27 °C. The conditioning process took at least 16 h. The temperature of the dope solution was controlled by temperature sensors. Hollow fibers were obtained using an annular spinneret with an outer/inner diameter of 1.7/0.8 mm. To form the lumen of the hollow fiber, a mixture of NMP/water (70/30 wt %) was fed inside. At the same time, distilled water was irrigated from the outside of the dope solution, i.e., the “wet air gap” option was used. The spinneret was equipped with the possibility of external irrigation. The take-up speed was selected each time in such a way as to exclude fiber drawing. The detailed spinning conditions and parameters are compiled in Table 2. After spinning, the samples of hollow fiber membranes were sequentially washed with tap water, then with ethanol for 2 h, then with n-hexane for 2 h, after which they were dried in air at room temperature.

### 2.5. Study of the Phase Inversion Kinetics at Different Temperatures

The phase inversion kinetics of dope solutions was studied by measuring the coagulation rate in a “limited” layer of a polymer solution [39]. This technique makes it possible to simulate the formation of a polymer membrane of a given thickness and to visualize structure formation in an asymmetric membrane. The cell consisted of two cover glasses glued with double tape, with a gap between the glasses of 100 μm and a channel depth of 300 μm. The channel was then filled with the polymer solution, and the whole assembly was fixed on a microscope slide; this slide was placed horizontally, normal to the optical axis of the microscope. The coagulant was added to the polymer solution using a Pasteur pipette from the side open to the atmosphere. Distilled water was used as a coagulant, which corresponds to the “wet air gap” regime used in this work. The cell with the dope solution was thermostatically controlled before measurements. The temperatures for measuring the phase inversion kinetics were the same as those used to prepare the hollow fiber membranes. The process of phase separation was observed normally to the cover glass using a Micromed R-1 optical microscope and was recorded on a digital camera (HiROCAM MA88, Premiere, Tonawanda, NY, USA). The phase inversion kinetics was estimated from the coagulation rate υ of the polymer solution layer, which is calculated as the ratio of the total thickness of the polymer layer (μm) to its coagulation time (s). The rate of the coagulation front was averaged over three measurements.

### 2.6. Gas Transport Properties

Helium and carbon dioxide were used as test gases, as their molecular mass difference provides a reliable way to determine Knudsen gas flow using the ideal selectivity value (the permeability coefficients of individual gases ratio). The pure gas permeance through hollow fiber membranes was measured using a volumetric membrane apparatus. The hollow fiber membrane was set inside the module. The gas flow was fed into the module on the external side of the hollow fiber. The volumetric gas flow passing through the membrane was measured using a dry gas meter (Shinagawa, Japan). Gas permeance measurements were carried out at room temperature (23 ± 1 °C) under transmembrane pressure from 0.5 to 2 bar, while permeate gas pressure was kept constant at 1 bar. Gas permeance was calculated using the equation:

(1) $$\frac{P}{l}=\frac{Q}{p\;\cdot \;S}$$

where *Q*—gas volumetric flow rate through the membrane, m^3^/h; *p*—transmembrane pressure, atm; *S*—membrane surface, m^2^. The gas volumetric flow rate was calculated using the equation:

(2) $$Q=\frac{V}{\tau }\;$$

where *V*—the volume of gas passed through the membrane, m^3^; *τ*—time for the specified gas volume transfer, h. The membrane area was calculated using the equation:

(3) $$S=\pi \;\cdot \;D_{\mathrm{out}}\;\cdot \;l$$

where D_out_—hollow fiber outer diameter, m; l—fiber length, m. Ideal selectivity was calculated using the equation:

(4) $$\alpha =\frac{\left(P_{1}/l\right)}{\left(P_{2}/l\right)}=\frac{P_{1}}{P_{2}}$$

where (*P*_1_/*l*)—He permeance, m^3^/(m^2^·h·atm); (*P*_2_/*l*)—CO_2_ permeance, m^3^/(m^2^·h·atm).

### 2.7. Porosimetry

The porosity of PSF hollow fiber membranes was determined using the instrument POROLIQ 1000 ML (POROMETER, Nazareth, Belgium). Membrane pore size analysis was performed by the liquid–liquid displacement method using water-saturated isobutanol and isobutanol-saturated water as a solvent pair. This technique allows the measurement of a pore size distribution from 2 to 500 nm. The porous structure was characterized by the diameter of the largest pore (d_max_) and the diameter of the smallest pore (d_min_), as well as the mean flow pore size d_MFP_ (MFP). The MFP value is defined as the pore size at which 50% of the flux penetrates through the larger pores and 50% of the flux penetrates through the smaller pores of the membrane skin layer. This technique also allows for determining the surface porosity ε, which is defined as the ratio of the area of the transport pores to the total surface area of the hollow fiber membrane.

### 2.8. Scanning Electron Microscopy

The membrane structure was studied by scanning electron microscopy (SEM) using a Hitachi «Tabletop TM 3030 Plus» microscope with a high-sensitivity low-vacuum secondary electron detector (Hitachi High Technologies Corporation, Tokyo, Japan). The accelerating voltage during image acquisition was 15 kV. The thickness of the gold layer was 5 nm.

## 3. Results and Discussion

### 3.1. Effect of Temperature on the Dope Solution Viscosity

To describe the temperature dependence of viscosity for non-Newtonian fluids, the well-known Arrhenius–Frenkel–Eyring equation is used [40,41]:

(5) $$\eta \;={\;\mathrm{Ae}}^{\frac{E}{\mathrm{RT}}}$$

where A—a pre-exponential coefficient that depends on the molecular nature and has the dimension of viscosity; E—the activation energy of viscous flow, J/mol; R—the gas constant, J/(mol∙K); and T—the absolute temperature, K.

**Figure 1:**
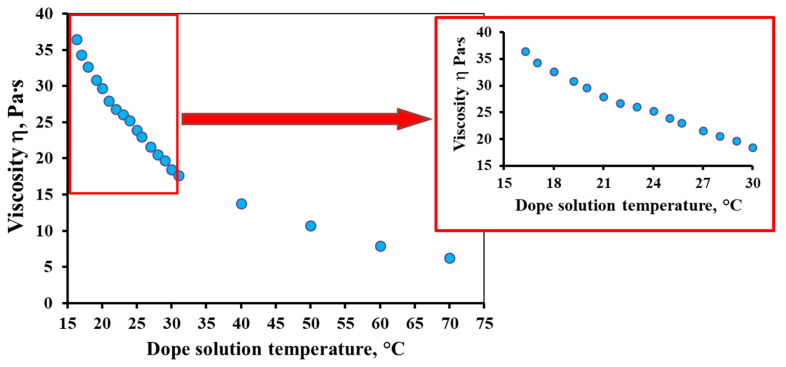
The temperature dependence of the dope solution viscosity.

Figure 1 shows the temperature dependence of the dope solution viscosity. The temperature range of 16–70 °C was chosen for this study, which includes the temperature range used in the experiments on membrane formation (17–27 °C). As can be seen from Figure 1, the decrease in viscosity with increasing temperature is exponential. In the studied temperature range, the dynamic viscosity of the dope solution decreases by almost six times from 36.5 to 6.3 Pa∙s. It should be emphasized that in the temperature range of 20–25 °C (“room temperature”), a significant change in viscosity can also be observed by approximately 5.8 Pa∙s (from 29.6 to 23.8 Pa∙s with an increase in temperature from 20 to 25 °C, respectively). This is about 20% in percentage terms. The change in viscosity in the temperature range used for the formation of PSF hollow fiber membranes in this work is 12.7 Pa∙s (decreases from 34.3 to 21.6 Pa∙s with an increase in temperature from 17 to 27 °C, respectively), which is approximately 37%.

As we can see, the temperature of the dope solution greatly affects the value of its viscosity. That is why this parameter cannot be neglected when obtaining hollow fiber membranes by the method of phase inversion induced by the contact with a non-solvent. Therefore, the indication of the temperature of the spinning process used in the literature as “room temperature” [16,29,30] does not seem to be entirely correct. This makes it difficult to reproduce the results obtained by other researchers based on data published in the open literature.

The mutual diffusion of solvent and non-solvent molecules in opposite directions from the polymer solution and into it, respectively, brings the system out of equilibrium, which leads to polymer coagulation. Viscosity and diffusion are interrelated properties that govern fluid dynamics. Therefore, it is well known that to calculate the diffusion coefficients of liquid molecules from viscosity and vice versa, the Stokes–Einstein relation is usually used. Obviously, a noticeable change in viscosity will significantly affect the phase inversion kinetics of the polymer solution. In this regard, a deviation in the temperature of the dope solution by only a few degrees can lead to the production of hollow fiber membranes with different properties from each other. For this reason, a very important stage is the study of the coagulation kinetics of the dope solution, depending on its temperature.

### 3.2. The Study of Phase Inversion Kinetics

**Figure 2:**
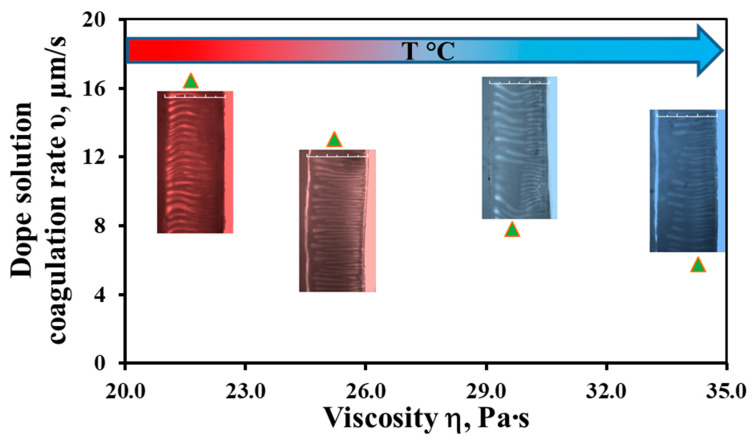
The dependence of the coagulation front rate on the viscosity of the PSF/NMP/PEG-400 dope solutions; the visualization of the solution structure during phase separation.

The study of the phase inversion kinetics was carried out in a “limited” layer [39] with a thickness of 300 μm. It is very important that the proposed method can monitor the process of phase inversion in real time and record it using a camera in an optical microscope. The results obtained are shown in Figure 2. The choice of distilled water as a coagulant is due to the fact that it was used as a non-solvent to form a skin layer on the outer surface of the hollow fiber in the “wet air gap” spinning regime.

The phase inversion kinetics was estimated from the passage rate of the coagulation front of the full thickness of the polymer solution layer. At the end of the movement of this front, only the primary polymer matrix is formed. Obviously, this is followed by the removal of the residual solvent and the final coagulation of the dope solution. The rate of the coagulation front has great importance in the process of hollow fiber membrane production. For example, it is necessary to know the time required for the primary mechanically stable framework formation of the polymer matrix of a certain thickness membrane before the resulting hollow fiber begins to be wound on a collection drum [38]. Depending on this time, it is possible to choose the required length of the coagulation path of the hollow fiber membrane in the coagulation bath.

Figure 2 shows that the rate of the polymer solution coagulation front changes by a factor of 2.8, from 16.5 to 5.8 μm/s, with a change in viscosity in the studied temperature range of 17–27 °C. As we can see, an increase in the dope solution viscosity hinders the mutual penetration of solvent molecules (NMP) and the non-solvent (distilled water) in opposite directions from the polymer solution and into it.

An important factor that affects the process of phase separation is the local polymer concentration in the film [42], which is determined as a function of the coordinate and time. This parameter allows for defining the type of phase separation process. Two types of phase formation processes are possible, leading to different types of membrane morphology: instantaneous liquid/liquid phase separation and delayed liquid/liquid phase separation. Instantaneous phase separation implies that the membrane is formed immediately after contact with the non-solvent, while in the case of delayed phase separation, some time elapses before the membrane formation. If the formation of the two liquid/liquid phases proceeds instantaneously, then, as a rule, membranes with a relatively porous skin layer are obtained. However, if the liquid/liquid phase separation begins after some time, membranes with a relatively dense skin layer are obtained [42]. The viscosity influence, and, hence, the phase inversion kinetics, on morphology, transport properties and pore structure are discussed in the following sections.

The study of the coagulation kinetics in a “limited” layer allows us to conclude that with an increase in the dope solution viscosity, the number of finger-like macrovoids, at first glance, remains almost unchanged, but their length decreases along the thickness of the resulting membrane. Nevertheless, some researchers [24,27] observed a transition in the obtained membrane structures from finger-like to sponge-like with an increase in the dope solution viscosity. However, with these structure changes, the concentration [24] and molecular weight [27] in the dope solution changed along with the viscosity.

The method used in this work makes it possible to predict the morphology of flat-sheet membranes with high accuracy [39]. At the same time, when creating hollow fiber membranes using the NIPS method, a bore fluid (in this work, NMP/water (70/30 wt %)) is fed into the polymer solution, so the coagulation (or the coagulation front movement) occurs towards each other from two sides of the membrane wall.

### 3.3. Influence of the Dope Solution Viscosity on Other Spinning Parameters

**Figure 3:**
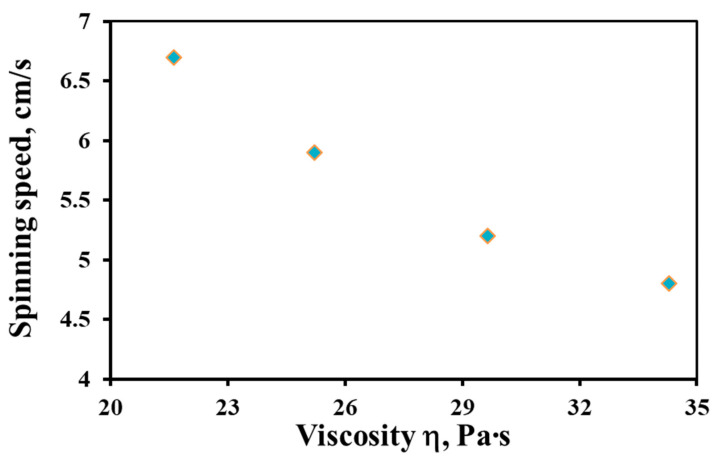
The speed dependence of a hollow fiber membrane spinning on the dope solution viscosity.

Before proceeding to the study of the properties of the obtained hollow fiber membranes, it is necessary to consider the influence of the dope solution viscosity on other parameters of the spinning process. Obviously, other parameters being constant (bore fluid flow rate, air gap, and extrusion pressure), the dope solution viscosity affects the spinning speed. This parameter is defined as the speed of the hollow fiber membrane spinning line. Figure 3 shows the dependence of the spinning speed on the dope solution viscosity. Figure 3 shows that with a decrease in the dope solution viscosity in the studied temperature range, the spinning speed increases by a factor of 1.4, from 4.8 to 6.7 cm/s.

Obviously, a change in the spinning speed will affect the residence time (RT) of the hollow fiber, which also depends on the size of the air gap and the rotation speed of the take-up drum [4,43]. In general, in the dry-jet wet spinning process, a long RT promotes the evaporation of the solvent from the outer surface of the polymer solution, as well as moisture penetration into the dope solution from the ambient atmosphere [44]. This leads to delayed phase separation, the formation of sponge-like structures, a decrease in the macrovoid size [44,45] and a decrease in gas permeance [46,47]. In our case, to exclude the effect of changes in the humidity of the ambient air, the process of hollow fiber membrane spinning was carried out with external irrigation with water.

A change in the dope solution viscosity can lead to a change in the draw ratio, which, apparently, largely determines the change in the coagulation speed (Figure 3). From the inner and outer sides of the hollow fiber, the same flow rate of the bore fluid and external coagulant was always supplied. Obviously, as the dope solution viscosity decreases, the nascent hollow fiber will be “drawn out” more and more by these fluid flows. The draw ratio is an important parameter that allows one to change the geometry and morphology of the obtained hollow fibers [4,38].

### 3.4. Properties of Hollow Fiber Membranes

Table 3 presents SEM microphotographs of the cross-section of the resulting hollow fiber membranes, as well as their inner and outer surfaces. It can be seen that all samples of hollow fiber membranes have an asymmetric structure with a thin skin layer and a porous support penetrated by finger-like macrovoids. SEM images make it possible to estimate the skin layer thickness of asymmetric hollow fiber membranes. There is a tendency towards its thinning (from 1.0 µm to 0.8 µm) with a decrease in the viscosity of the spinning solution (from 34.3 to 21.6 Pa s). It can be noted that there are no visible changes in the morphology of the inner and outer surfaces with a viscosity change. Additionally, there is no noticeable change in the geometric dimensions of the finger-like macrovoids. It is difficult to estimate their lengths due to the variation in the wall thickness of the resulting hollow fiber membranes.

**Figure 4:**
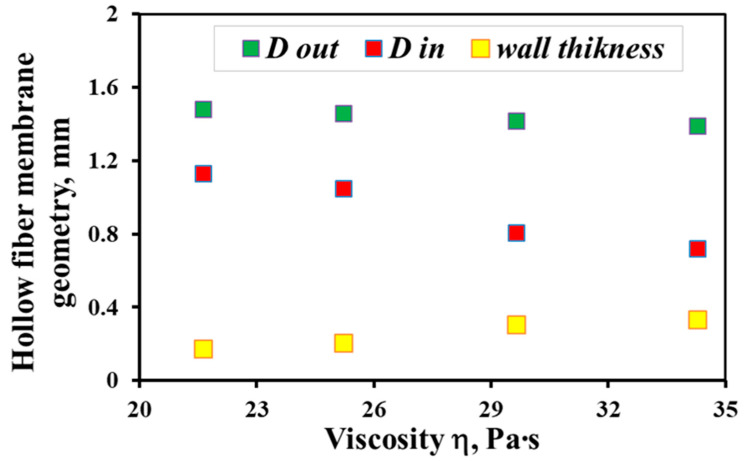
The dependence of the geometric parameters of the hollow fiber membrane on the dope solution viscosity.

By analysis of the SEM images, the geometrical parameters of the hollow fiber, such as the average outer and inner diameters (D_out_ и D_in_), and membrane wall thickness were estimated. The results obtained are shown in Figure 4. With a decrease in the dope solution viscosity, the wall thickness of the hollow fiber decreases with a simultaneous increase in both the outer and inner diameters. Indeed, at fixed values of the dope solution pressures and bore fluid feed rate, a decrease in the viscosity of the solution results in an increase in the rate of its outflow from the spinneret. Therefore, a constant bore fluid feed rate and an increase in the polymer solution feed rate—due to a lower viscosity—results in an increase in the hollow fiber internal diameter because the bore fluid pressure under these conditions expands the fiber walls, simultaneously reducing their wall thickness [48,49,50]. As can be seen from Figure 4, in the temperature range under study, the wall thickness of the hollow fiber membrane decreases by almost a factor of 2, from 340 µm at 17 °C to 180 µm at 27 °C.

**Figure 5:**
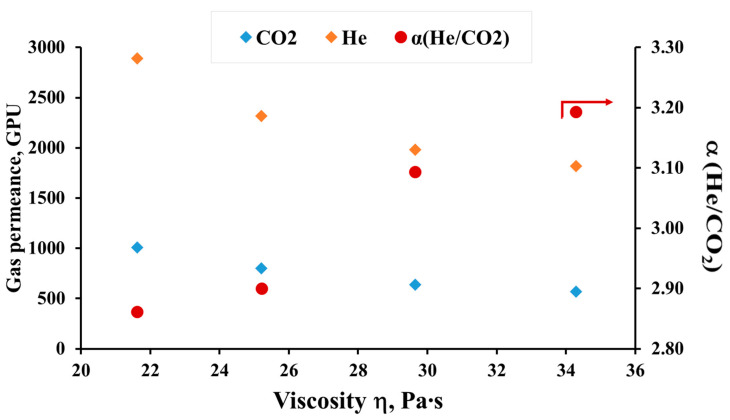
Transport properties of PSF hollow fiber membranes depending on the dope solution viscosity. 1 GPU = 10^−6^ cm^3^[STP] cm^−2^ s^−1^ cmHg^−1^.

Figure 5 shows data on the gas permeance and ideal selectivity of the He/CO_2_ gas pair of PSF hollow fiber membranes obtained from the dope solutions of the same composition but at different temperatures. It can be seen that a decrease in the viscosity of the solutions leads to the production of hollow fiber membranes with higher gas permeance values. The permeance for individual He and CO_2_ gases with decreasing viscosity in the studied temperature range increases by about 1.6–1.8 times (from 1820 to 2890 GPU and from 570 to 1010 GPU for He and CO_2_, respectively). In this case, selectivity decreases: for hollow fiber membranes obtained from dope solutions at 17 °C *α* (He/CO_2_) is 3.19 and at 27 °C is 2.86. It should be noted that the values of the ideal selectivity for the He/CO_2_ gas pair indicate that in the obtained membranes, gas transport is realized that is close to the Knudsen gas flow regime. Thus, in the Knudsen gas flow regime, the ideal selectivity for the He/CO_2_ gas pair is 3.3. The obtained results on gas permeance generally agree with the literature data [13,14,16,17], where, with an increase in the polymer concentration in the dope solution and, hence, its viscosity, a decrease in gas permeance was observed with a simultaneous increase in selectivity. The authors explain this by a slowdown in the kinetics of phase separation, which contributes to an increase in skin layer thickness [15], a decrease in porosity and the transport pore size [14,16].

**Figure 6:**
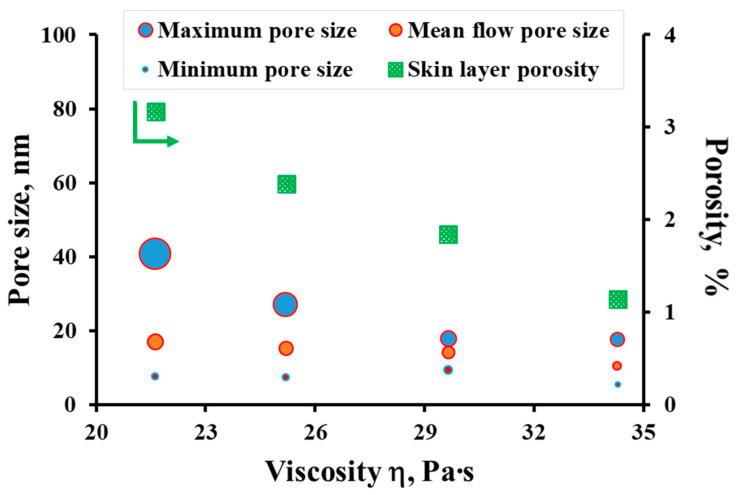
The dependence of the pore size and porosity of PSF hollow fiber membranes on the dope solution viscosity.

To evaluate the porous structure parameters of PSF hollow fiber membranes, the following values were determined: the sizes of the largest and smallest pores, the mean flow pore size and the surface porosity *ε*. The dependence of these values on the dope solution viscosity is shown in Figure 6. The obtained results on the evaluation of the pore size correlate with the data obtained on the gas permeance of the investigated PSF hollow fiber membranes. With a decrease in the dope solution viscosity in the temperature range of 17–27 °C, the mean flow pore size of the PSF hollow fiber membranes increases from 10.4 to 17.0 nm. The surface porosity also increases by about three times. The results obtained also coincide with the literature data [17,19,23,24], where the dope solution viscosities were changed by changing their compositions. There is also a relationship between the values of ideal selectivity and the maximum pore size of the membranes, which has a significant effect on the separating properties of the obtained PSF hollow fiber membranes. It is worth noting the narrow pore distribution of the hollow fiber membranes (the minimum pore size is 5.6 nm; the maximum pore size is 40.6 nm). This circumstance makes these membranes promising for use as supports for producing gas separation composite hollow fiber membranes with selective layers, for example, from the promising polymer polydecylmethylsiloxane. This polymer has proven to be excellent for the extraction of C_3+_ hydrocarbons from a mixture with methane [51,52].

The obtained data on the influence of the viscosity of the PSF/NMP/PEG-400 dope solutions, which was changed by its temperature, on the properties of hollow fiber membranes are given for convenience in the summary Table 4.

## 4. Conclusions

For the first time, the influence of the dope solution viscosity of a constant composition on the properties of PSF hollow fiber membranes was studied. The viscosity of the dope solution and the spinning mode were varied by changing the temperature. The change in the dope solution temperature was carried out in a rather narrow temperature range of 17–27 °C, corresponding to the spinning modes at so-called “room temperature”. Using the PSF/NMP/PEG-400 dope solutions as an example, it was shown that this parameter cannot be neglected when obtaining hollow fiber membranes by the NIPS method. The indication of the temperature of the spinning process as “room temperature” used in some studies does not seem to be entirely correct since it makes it difficult to reproduce the results obtained by other researchers. It has been shown that an increase in temperature and, consequently, a decrease in the viscosity of the PSF/NMP/PEG-400 dope solutions lead to the following:

- The rate of the polymer solution coagulation front changes by a factor of 2.8, from 16.5 to 5.8 cm/s;
- The speed of the hollow fiber membrane formation increases 1.4 times, from 4.8 to 6.7 cm/s, and the draw ratio also increases;
- The thickness of the skin layer decreases from 1.0 to 0.8 µm; the wall thickness of the hollow fiber membrane decreases from 340 µm to 180 µm with a simultaneous increase in both the outer and inner diameters;
- The gas permeance for individual gases He and CO_2_ increases 1.6–1.8 times, from 1820 to 2890 GPU and from 570 to 1010 GPU, respectively, while the selectivity decreases from 3.19 to 2.86;
- The mean flow pore size of hollow fiber membranes increases from 10.4 to 17.0 nm, the surface porosity also increases by about three times.

## Figures and Tables

**Table 1:** Influence of the change in the spinning solution viscosity, caused by various reasons, on the properties of hollow fiber membranes (literature data).

Pol.	Solv.	Add.	Method	Way to Viscosity Change	Effect of Viscosity Increasing	Application	Ref.
PSF	DMAc/THF	Ethanol	NIPS	Polymer concentration **↗**	Gas permeance **↘** Gas selectivity **↗**	HF support	[13]
PEI	NMP	-	NIPS	Polymer concentration **↗**	N_2_ permeance, surface porosity, pore size, hydrophobicity **↘**	CO_2_ absorption	[14]
ECTFE	DEP, GTA	-	TIPS	Polymer concentration **↗**	Skin layer thickness, tensile strength **↗** Water permeability **↘**	MF	[15]
PES	NMP	-	NIPS	Polymer concentration **↗**	Pore size, surface porosity, He permeance **↘**	CO_2_ absorption	[16]
PEI	NMP	-	NIPS	Polymer concentration **↗**	Skin layer thickness, gas selectivity **↗** Gas permeability **↘**	HF support	[17]
PVDF	DPC	-	TIPS	-Polymer concentration **↗** -Polymer M_w_ **↗**	Water permeability, pore size **↗**	UF	[18]
PES/sPSF	NMP	LiBr	NIPS	-Ratio of polymers -Polymer concentration **↗**	Surface porosity, water permeability, MWCO **↘**	NF	[19]
PBT	NMP	PEG-6000	NIPS	-Polymer concentration **↗** -Additive concentration **↗**	Porosity, pore size, water permeability **↘**	UF	[20]
PVP/PVC	NMP/THF	-	NIPS	Polymer weight ratio	Porosity, water permeability, MWCO **↘** Salt rejection **↗**	Forward osmosis	[21]
CPES/PES	DMAc	PEG-200	NIPS	CPES content in total polymer concentration **↗**	Hydrophilicity, water permeability **↗** Tensile strength, rejection **↘**	UF	[22]
PES	NMP, DMAc	Ethanol, glycerol, PVP	NIPS	Additive types	Tensile strength, water permeability **↗** Rejection **↘**	NF	[23]
PVDF	NMP	H_2_O	NIPS	-Additive concentration **↗** -Storing in closed vessels **↗**	Structure: finger-like→sponge-like Porosity, pore size **↗**	Membrane distillation	[24]
PVDF	DMAc/TEP	PVP, SiO_2_	NIPS	Additive (SiO_2_) concentration **↗**	Breaking strength, Young’s modulus, water permeability, rejection **↗**	Vacuum membrane distillation	[25]
PVDF	NMP	LiCl	NIPS	Additive concentration **↗**	Microvoid size, N_2_ permeance, pore size, overall porosity **↘**	CO_2_ absorption	[26]
PVDF	NMP	PEG	NIPS	Additive M_w_ **↗**	Structure: finger-like→sponge-like N_2_ permeance **↗**	Air filtration	[27]
PVDF	Triacetin	-	TIPS	Extrusion temperature (140–170 °C)	Pore size, hydrophobicity, membrane strength, porosity, CO_2_ flux **↘**	CO_2_ absorption	[28]

PVDF—polyvinylidene fluoride; PEG—polyethylene glycol; PES—polyethersulfone; CPES—carboxylic polyethersulfone; sPSF—sulfonated polysulfone; PVP—polyvinylpyrrolidone; PEI—polyethylenimine; EG—ethylene glycol; PVC—polyvinylchloride; PBT—poly(biphenyl-trifluoroacetone); ECTFE—poly(ethylene-chlorotrifluoroethylene); TEP—triethylphosphate; THF—tetrahydrofuran; NMP—N-methyl-2-pyrrolidone; DMAc—dimethylacetamide; DEP—diethyl phthalate; GTA—glycerol triacetate; DPC—diphenyl carbonate; MWCO—molecular weight cut-off; and HF—hollow fiber.

**Table 2:** Spinning parameters for PSF hollow fiber membranes.

Parameters	
Dope solution composition (PSF/PEG-400/NMP, wt %)	22/30/48
Dope solution temperature (°C)	17, 20, 24, 27
Extrusion pressure (atm)	5
External coagulant	Tap water
Coagulant bath temperature (°C)	20 ± 1
Bore fluid type	NMP/water (70/30 wt %)
Bore fluid temperature (°C)	20 ± 1
Bore fluid flow rate (mL/min)	3.5 ± 0.3
Spinneret dimension (mm)	OD/ID = 1.7/0.8
Wet air gap (cm)	50

**Table 3:** SEM microphotographs of PSF hollow fiber membranes obtained at different dope solution temperatures.

T, °C	Viscosity, Pa · s	Cross-Section	Inner Surface	Outer Surface
17	34.3	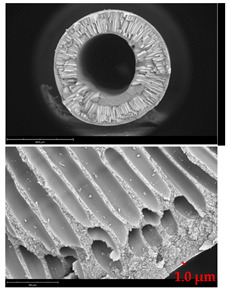	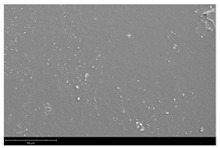	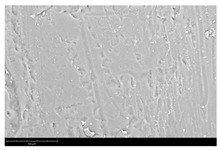
20	29.6	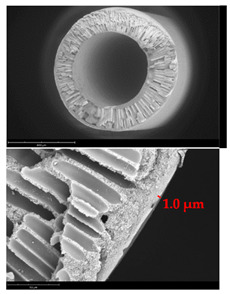	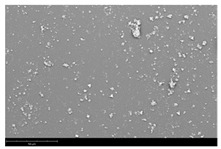	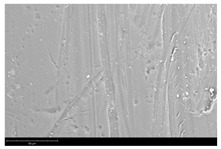
24	25.2	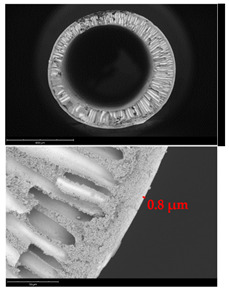	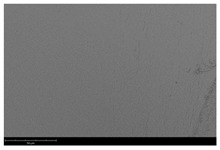	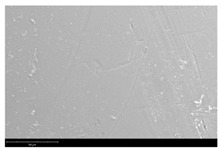
27	21.6	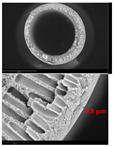	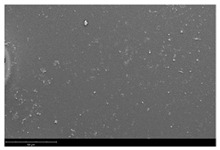	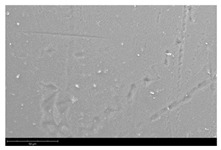

**Table 4:** Summary table with the obtained data on the influence of the dope solution viscosities on the properties of hollow fiber membranes.

T, °C	η, Pa∙s	υ, µm/s	D_out_, mm	Wall Thickness, mm	P/l (CO_2_), GPU	α (He/CO_2_)	d_max_, nm	d_MFP_, nm	ε,%
**17**	34.3	5.76	1.39	0.34	570	3.19	17.7	10.4	1.14
**20**	29.6	7.79	1.42	0.31	640	3.09	17.9	14.3	1.84
**24**	25.2	13.05	1.46	0.21	800	2.90	27	15.3	2.39
**27**	21.6	16.45	1.48	0.18	1010	2.86	40.8	17	3.17

(1 GPU = 10^−6^ cm^3^[STP] cm^−2^ s^−1^ cmHg^−1^).

## Data Availability

Not applicable.
